# Announcement

**Published:** 1892-01

**Authors:** 


### ANNOUNCEMENT.

With this issue we make a change in our editorial staff, Dr. Wm. Perrin Nicolson and Dr. F. O. Stockton retiring, and the places which have been so ably filled by them will in future be supplied by Dr. J. McFadden Gaston and Dr. Willis F. Westmoreland.

To the profession Dr. Gaston needs no introduction. As Professor of the Practice and Principles of Surgery in the Southern Medical College, as a contributor to Wood's Reference Hand-Book, as one of the editorial staff of the Annual of the Universal Medical Sciences and as a contributor to the International Clinic, he has made for himself a national fame. He comes to us bringing with him a rich store of knowledge which he has acquired through long years of the practice of his branch, both in this and other countries. He is also Chairman of the Section of Surgery of the American Medical Association and President of the Southern Surgical and Gynæcological Association.

The mere name of Westmoreland is too well known in medical circles, both North and South, to need mention here. Dr. Westmoreland is the brilliant young Professor of Surgery in the Atlanta Medical College, is a gentleman of high scholastic attainments, and is a bright luminary among the foremost medical writers of the day.

We are determined during the year 1892 to get out a journal that will, both for selection of matter published and artistic typographical design, take a high stand among the very few really excellent ones which are now published—one especially adapted to the active practitioner, and which will give him in a concise manner all the leading and valuable medical news of this progressive age.

It was our intention to change our cover with this issue, but being unable to get paper cannot do so till our next.

---

MEMBERSHIP in the American Pharmaceutical Association is obtained only by election at the annual meeting. "Every pharmacist and druggist of good moral and professional standing, whether in business on his own account, retired from business, or employed by another, and those teachers of pharmacy, chemistry and botany who may be especially interested in pharmacy and materia medica" are eligible for membership. For blank application and further information, address Dr. H. M. Whelpley, 2729 Washington Avenue, St. Louis, Mo., Chairman of the Committee on membership.

